# 2P2Idb v2: update of a structural database dedicated to orthosteric modulation of protein–protein interactions

**DOI:** 10.1093/database/baw007

**Published:** 2016-03-15

**Authors:** Marie-Jeanne Basse, Stéphane Betzi, Xavier Morelli, Philippe Roche

**Affiliations:** Centre de Recherche en Cancérologie de Marseille (CRCM); CNRS, UMR 7258; INSERM U1068; Institut Paoli-Calmettes; Aix-Marseille Université; Marseille 13009, France

## Abstract

2P2Idb is a hand-curated structural database dedicated to protein–protein interactions with known small molecule orthosteric modulators. It compiles the structural information related to orthosteric inhibitors and their target [i.e. related 3D structures available in the RCSB Protein Data Bank (PDB)] and provides links to other useful databases. 2P2Idb includes all interactions for which both the protein–protein and protein–inhibitor complexes have been structurally characterized. Since its first release in 2010, the database has grown constantly and the current version contains 27 protein–protein complexes and 274 protein–inhibitor complexes corresponding to 242 unique small molecule inhibitors which represent almost a 5-fold increase compared to the previous version. A number of new data have been added, including new protein–protein complexes, binding affinities, molecular descriptors, precalculated interface parameters and links to other webservers. A new query tool has been implemented to search for inhibitors within the database using standard molecular descriptors. A novel version of the 2P2I-inspector tool has been implemented to calculate a series of physical and chemical parameters of the protein interfaces. Several geometrical parameters including planarity, eccentricity and circularity have been added as well as customizable distance cutoffs. This tool has also been extended to protein–ligand interfaces. The 2P2I database thus represents a wealth of structural source of information for scientists interested in the properties of protein–protein interactions and the design of protein–protein interaction modulators.

**Database URL:** http://2p2idb.cnrs-mrs.fr

## Introduction

Protein–protein interactions (PPIs) are becoming more and more recognized therapeutic targets due to their central role in biological pathways and in physiopathological processes (for reviews, see Refs 1–4). However, PPIs have long been considered as poorly druggable targets leading to low success rates in drug discovery campaigns. Over the last 15 years, a better characterization of the protein–protein interfaces together with the conception of PPI-oriented chemical libraries has facilitated the development of new small-molecule PPI-inhibitors and PPI-stabilizers for an increasing number of targets (for reviews, see Refs 4–12). Most PPI modulators compete directly with the protein–protein interface (orthosteric modulators) by targeting key ‘hot spots’ residues responsible for the majority of the binding free energy (13, 14) or by mimicking secondary structure elements from the protein partner such as alpha helices or beta strands (15, 16). PPI modulators can also bind away from the interface (allosteric inhibition) often involving structural and/or dynamic changes over the target (4, 17). Although both types of inhibitors can be successful in the design of chemical probes or drugs, allosteric inhibition is more difficult to predict *in silico* and a number of compounds have been identified serendipitously (4, 18). Therefore, we decided to concentrate only on orthosteric inhibition. To improve the discovery of new orthosteric PPI modulators, it is important to uncover the basic principles underlying the mechanisms of protein–protein and protein–ligand recognition and to characterize the properties of known PPI disruptors. To that extend, and in an effort to organize and analyze the fast growing amount of structural data available, we have developed 2P2Idb, a hand-curated structural database dedicated to orthosteric modulation of PPIs that was first released in 2010 (19, 20). The 2P2I structural database holds information on protein–protein and protein–ligand complexes that have been structurally characterized and available in the RCSB Protein Data Bank (21, 22). Two other resources focusing on the properties of PPI modulators are available: TIMBAL (23, 24) and iPPI-DB (25). TIMBAL contains >8000 PPI modulators automatically retrieved from the ChEMBL database, whereas iPPI-DB contains 1650 non-peptide inhibitors across 13 families of PPIs. The chemical structures, the physicochemical and the pharmacological profiles of the inhibitors in iPPI-DB are manually extracted from the literature. There are two major differences between 2P2Idb and the other two databases, i/2P2Idb is a structural database dedicated to PPI modulators with structural information for both the protein–protein and protein–ligand complexes as well as for the small-molecule compounds and ii/2P2Idb focuses only on orthosteric inhibitors (directly interfering at the interface). These differences account for the limited number of entries in 2P2Idb compared to the other two databases. Over the last 5 years, the database has grown constantly leading to a 5-fold increase in the number of small molecule compounds. Conclusions from the analysis of 2P2Idb have already proved useful to characterize PPI inhibitors and to guide the development of tools to filter large chemical libraries in order to build PPI-oriented chemical libraries (8, 26, 27).

## Materials and Methods

### 2P2Idb

2P2Idb is a relational database that was built through data mining from literature and by exhaustive search of the Protein Data Bank (http://www.rcsb.org/). The list of 27 protein–protein complexes was retrieved through searches in the PDB, literature or conferences abstracts. Homology models were built with Modeller package v9.14 (28) for protein–protein complexes without a 3D structure (bromodomains BRD3-1, BRD3-2, BRD4-2, BRDT-1 and KRAS) using a close homolog (identity ranging from 75% to 94%) as a template.

The database is updated through an automatic process with several checkpoints. For each protein–protein complex, a list of PDB codes is retrieved from Uniprot (http://www.uniprot.org) and new protein–ligand complexes are detected and superimposed onto the equivalent protein–protein complex. A pymol file is automatically generated and a manual examination is performed to ensure that small molecule compounds are binding at the protein–protein interface (covalently bound ligands are not included in the database). Once validated, PDB files are formatted and a series of in-house PhP and Perl scripts are used to automatically retrieve the necessary data to fill the database (such as Pubmed Id, Chain and Ligand 3-Character code). Binding affinity data are retrieved form PDBbind v2015 (http://www.pdbbind-cn.org/) or BindingDB (http://www.bindingdb.org/) or BindingMOAD (http://www.bindingmoad.org/). Small molecule ligands are retrieved from the PDB as SDF files. ChemSpider Ids are automatically retrieved using a Perl script adapted from resources available on the ChemSpider website (http://www.chemspider.com/). SDF files are standardized and formatted with MOE (https://www.chemcomp.com/) and Chemaxon (https://www.chemaxon.com). Molecular descriptors are computed with MOE and DRAGON (http://www.talete.mi.it/).

### 2P2I-inspector

Interface properties are calculated using a combination of in-house shell and tcl scripts and procedure in the VMD package version 1.9.2 (http://www.ks.uiuc.edu/Research/vmd/). Missing hydrogen atoms are added with Pymol (https://www.pymol.org/). Gap volumes, planarity, eccentricity and circularity are computed using SURFNET (29).

## Results and Discussion

### 2P2Idb update

**Figure 1:**
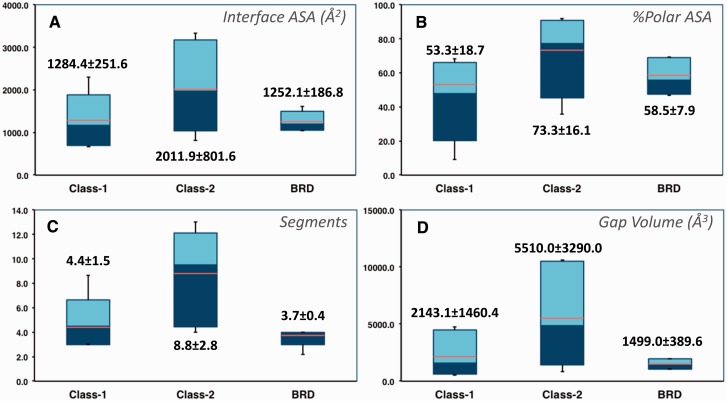
Selected characteristics of protein–protein interfaces for the three classes of protein–protein complexes. (**A**) Buried accessible surface area in Å^2^ (corresponding to the size of the interface). (**B**) Percentage of polar accessible surface area. (**C**) Total number of segments at the interface. (**D**) Gap volume at the interface in Å^3^ (corresponding to the volume enclosed between the two protein partners). The thin bars represent the complete range distribution for a given property (from minimum to maximum values) and boxes correspond to 90% of the complexes (from 5% to 95% of the distribution). Median values are shown through a color change from dark to light blue, whereas average values are indicated as orange lines. Average values and standard deviations are also shown explicitly for each distribution.

The 2P2I database focuses on orthosteric small molecule inhibitors of PPI, we therefore only select the cases for which both the protein–protein and protein–inhibitor complexes are present in the RCSB Protein Data Bank and for which the inhibitor is directly competing at the interface. The current release of the database contains 27 protein–protein complexes, 274 protein–inhibitor complexes, 28 free proteins and 242 unique small molecule modulators (Table 1). The protein–protein complexes were subdivided into three classes corresponding to protein–peptide complexes (class 1), protein–protein complexes with globular proteins (class 2) and bromodomains–histone complexes (class BRD) based on the total number of segments at the interface and target properties. Characteristics of the three types of protein–protein interfaces were computed with our local server tool (http://2p2idb.cnrs-mrs.fr/2p2i_inspector.html) and four selected properties are highlighted in Figure 1. Protein–protein complexes belonging to the bromodomain class exhibit properties similar to class 1 complexes; however, the specific mode of binding of these bromodomain targets recognizing and binding to acetylated lysine residues on histone substrates prompted us to define a different class for these targets. Indeed, compounds inhibiting the bromodomain targets are notably smaller and less hydrophobic (with lower LogP values) than those inhibiting the other two classes. On average protein–protein interfaces from class 2 are larger and more polar than interfaces from the other classes (Figure 1A and B). They are composed of 8.8 ± 2.8 segments versus 4.4 ± 1.5 and 3.7 ± 0.4 for interfaces from class 1 and bromodomains, respectively (Figure 1C). The gap volume corresponding to the volume enclosed between two interacting proteins is bigger on average for class 2 complexes (Figure 1D). Overall, as expected, the bromodomain class represents a more homogenous class than the other two classes for which great disparities can be observed from one complex to another.

**Table 1:** List of 27 protein–protein complexes in 2P2I database

**Family**	**Class**^a^	**PDB**^b^	**Uniprot**^c^	**Number of Inhibitors**^d^
BRD2-1/H4	BRD	BD21*	P25440	15
BRD2-2/H4	BRD	2E3K	P25440	7
BRD3-1/H4	BRD	BD31*	Q15059	1
BRD3-2/H4	BRD	BD32*	Q15059	1
BRD4-1/H4	BRD	3UVW	O60885	76
BRD4-2/H4	BRD	BD42*	O60885	1
BRDT-1/H4	BRD	BDT1*	Q58F21	2
Bcl2/Bax	1	2XA0	P10415	10
BclXL/Bak	1	1BXL	Q07817	20
CIAP1_1/CASPASE-9	1	3D9T	Q13490	10
CIAP1_2/SMAC	1	3D9U	Q13490	10
HDM2/P53	1	1YCR	Q00987	32
HPV_E2/E1	2	1TUE	P06790	1
HRAS/SOS1	2	1BKD	P01112	1
IL-2/IL-2R	2	1Z92	P60568	5
Integrase/LEDGF	2	2B4J	P12497	4
KEAP1/NRF2	2	2FLU	Q14145	9
KRAS/SOS1	2	KRAS*	P01116	19
MDM4/P53	1	3DAB	O15151	1
Menin/MLL	1	4GQ6	O00255	8
TNFR1A/TNFB	2	1TNR	P19438	1
TNFalpha	2	1TNF	P01375	1
VHL/HIF1A	2	4AJY	P40337	18
XDM2/P53	1	1YCR	P56273	11
XIAP/CASPASE-9	2	1NW9	P98170	16
XIAP/SMAC	1	1G73	P98170	16
ZIPA/FTSZ	1	1F47	P77173	4

^a^ Protein–protein complex class as defined in the text.

^b^ PDB code of the protein–protein complex (*denotes a homology model built with Modeller).

^c^ UNIPROT id of the target protein.

^d^ Number of small molecule inhibitors for the given target.

### 2P2Idb web interface

**Figure 2:**
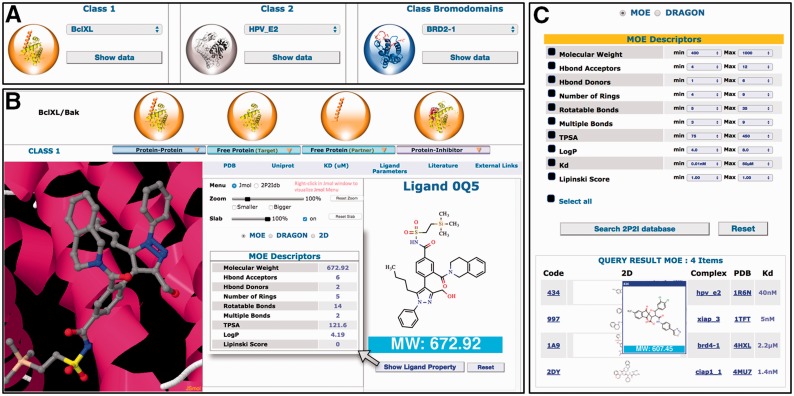
Overview of the 2P2Idb interface. (**A**) Protein–protein complexes divided into three subclasses can be selected using dropdown menus. (**B**) PDB structures corresponding to protein–protein complexes, free forms and protein–inhibitor complexes can be easily selected and visualized in a JSmol applet. All information included in the database including pre-calculated interface parameters, small compounds molecular descriptors and links to various websites of interest can be accessed. (**C**) Ligands in the 2P2I database can been searched by chemical properties. The range values of standard molecular descriptors can be defined to retrieve compounds from the database with matching properties.

A complete new web interface has been designed with new contents and new features (Figure 2). All 3D structures and related data for a given PPI family can now be accessed on the same web page. New tools and menus have been added to visualize the different 3D structures through a javascript JSmol applet. More information can be found about protein–protein complexes, protein–ligand complexes, free proteins and small molecule orthosteric modulators. These new data include non-bonded contacts, interfacial hydrogen bonds and salt bridges, geometrical, physicochemical parameters of the protein–protein interfaces, binding parameters and standard molecular descriptors for the small molecule compounds. Links to relevant websites and databases are provided such as literature (PubMed, DOI), proteins information (UniProt), 3D structures (PDBsum, RCSB, PDBe), ligand properties (ChemSpider, LigandExpo, CREDO), protein–protein and protein–lig and binding affinities from the latest version of PDBbind (30), BindingMOAD (31, 32) or BindingDB (http://www.bindingdb.org/). A large number of pre-calculated interface parameters are accessible for each protein–protein complex. These interface descriptors include total interface surface area, geometrical parameters (gap volume, planarity, eccentricity, circularity, number of segments) and type of interaction (non-bonded contacts, percentage of charged residues, hydrogen bonds, salt bridges, disulfide bonds, secondary structure). When there are several inhibitors for the same protein–protein complex, the different 3D structures of the protein–ligand complexes can be easily superimposed to compare the mode of binding of the inhibitors.

### 2P2I-inspector: a tool to analyze a protein–protein and protein–ligand interfaces

We have recently developed 2P2I-inspector, a web interface to calculate a series of interface parameters of protein–protein complexes (19). We have now extended this tool to protein–ligand complexes. 2P2I-inspector computes a large number of protein–protein and protein–ligand interface parameters from the 3D structure of the complex using a combination of in-house scripts, VMD (33) and SURFNET (29). New geometrical parameters including planarity, eccentricity and circularity of the interface have been included in the latest version. In addition, new optional menus have been developed to allow the user to modify default cutoff values used to compute interface properties such as non-bonded contacts, hydrogen bonds, salt bridges and number of segments thus providing a more flexible and versatile tool.

In conclusion, the freely accessible 2P2I website provides valuable structural information about the modulation of PPIs with orthosteric inhibitors. The database and its associated tools to calculate parameters for protein–protein and protein–ligand interfaces provide a wealth of high quality data that can be used to characterize protein–protein and protein–ligand interfaces and to improve and accelerate the design of protein–protein modulators.
